# American Gastroenterological Association-Proposed Fecal Calprotectin Cutoff of 50 ug/g is Associated With Endoscopic Recurrence in a Real-World Cohort of Patients With Crohn’s Disease Post-ileocolic Resection

**DOI:** 10.1093/crocol/otae016

**Published:** 2024-03-09

**Authors:** Terry Li, Ravi Shah, Benjamin Click, Benjamin L Cohen, Edward Barnes, Abel Joseph, Salam Bachour, Jessica Hu, Susell Contreras, Elizabeth Li, Jordan Axelrad

**Affiliations:** Department of Internal Medicine, NYU Grossman School of Medicine, New York, NY, USA; Department of Gastroenterology, Hepatology and Nutrition, Digestive Disease and Surgery Institute, Cleveland Clinic, Cleveland, OH, USA; Division of Gastroenterology and Hepatology, University of Colorado Anschutz Medical Campus, Aurora, CO, USA; Department of Gastroenterology, Hepatology and Nutrition, Digestive Disease and Surgery Institute, Cleveland Clinic, Cleveland, OH, USA; Division of Gastroenterology and Hepatology, University of North Carolina, Chapel Hill, NC, USA; Department of Internal Medicine, Cleveland Clinic, Cleveland, OH, USA; Department of Internal Medicine, Brigham and Women’s Hospital, Boston, MA, USA; University of North Carolina School of Medicine, ChapelHill, NC, USA; Division of Gastroenterology and Hepatology, NYU Grossman School of Medicine, New York, NY, USA; NYU Grossman School of Medicine, NewYork, NY, USA; Division of Gastroenterology and Hepatology, NYU Grossman School of Medicine, New York, NY, USA

**Keywords:** Crohn’s disease, ileocolic resection, fecal calprotectin, postoperative monitoring

## Abstract

**Background:**

Fecal calprotectin (FC) is a reliable predictor of active bowel inflammation in postoperative Crohn’s disease (CD), but cutoffs vary between studies. Recent guidelines recommend a cutoff of <50 ug/g to avoid routine endoscopy in patients at low pretest probability for CD recurrence. We evaluated the performance of this threshold in a real-world CD cohort after ileocolic resection (ICR).

**Methods:**

In this retrospective study, patients with CD post-ICR between 2009 to 2020 with FC > 60 days but < 1 year of surgery were included from a multicenter database. Established risk factors and/or biologic prophylaxis (biologic within 90 days of surgery) defined pretest probability. Those without postoperative colonoscopy were excluded. Rates of endoscopic recurrence, defined as Rutgeerts score ≥ i2b at any time after surgery, were compared between FC < 50 versus  ≥ 50 ug/g. Student’s *t*-test and Fisher’s exact test were utilized for statistical analysis. All postoperative FCs were matched to closest colonoscopy within 1 year to calculate sensitivity, specificity, positive predictive value (PPV), and negative predictive value (NPV).

**Results:**

Thirty-seven patients categorized as either low-risk or high-risk and received biologic prophylaxis and had postoperative colonoscopy were included. Median time to first FC was 217 days (IQR 131–288). 15 (41%) patients had initial FC < 50 ug/g versus 22 (59%) ≥50 ug/g. Median time to first colonoscopy was 234 days (IQR 189–369). Compared to initial FC ≥ 50 ug/g, FC < 50ug/g experienced less endoscopic recurrence (0% vs. 36%, *P* = .005). Median time to first endoscopic recurrence in FC ≥ 50 ug/g was 145 days. There were 39 matched pairs of FC and colonoscopy. At an FC cutoff of 50 ug/g, calculated sensitivity was 90% and NPV was 93%, whereas specificity and PPV were 48% and 38%, respectively.

**Conclusions:**

In this real-world cohort, FC < 50 ug/g is a useful cutoff to exclude endoscopic recurrence in a post-ICR CD population that is at low pretest probability of recurrence.

## Teaser

This retrospective cohort study of patients with Crohn’s disease after ileocolic resection assesses the real-world performance of guideline-proposed fecal calprotectin cutoffs to avoid routine endoscopy in patients at low pretest probability for recurrence.

## Introduction

Patients with moderate to severe Crohn’s disease (CD) often require surgery for medically refractory or complicated disease. Optimization of monitoring strategies after surgically induced remission is an area of active investigation. Fecal calprotectin (FC) is a noninvasive biomarker that can reliably predict active bowel inflammation^1^ and has been proposed as the foundation for algorithmic approaches to disease monitoring.^2,3^ However, the optimal cutoff in the postoperative CD population is debated. Existing studies have utilized different cutoffs that demonstrate variable sensitivity and specificity for disease recurrence.^4^ Recent American Gastroenterological Association (AGA) guidelines propose a cutoff of <50 ug/g to avoid routine endoscopy in patients at low pretest probability for recurrence based on a false negative rate of 1.4%.^5^ A cutoff of <150 ug/g in low-risk patients on prophylaxis is also suggested. To provide real-world corroboration, we assessed the performance of the guideline-proposed FC cutoffs in a multicenter cohort of patients with CD after ileocolic resection (ICR).

## Methods

This was a retrospective study of patients with CD after ICR using a database from 3 academic institutions spanning from 2009 to 2020. Patients with FC at least 60 days after but within one year of surgery were included. Established risk factors (surgery age, smoking, penetrating phenotype, and number of CD surgeries) and/or biologic prophylaxis (biologic within 90 days of surgery) were used to define the risk for recurrence. Those without postoperative colonoscopy were excluded. Rates of endoscopic and surgical recurrence were compared between FC < 50 versus  ≥ 50 ug/g. Endoscopic recurrence was defined as Rutgeerts ≥ i2b,^6^ determined by the endoscopist during assessment or, if unavailable, by retrospective review of scope images by a gastroenterologist, at any point after surgery. Student’s *t*-test and Fisher’s exact test were utilized for statistical analysis. All FCs were matched to closest colonoscopy within 1 year to calculate sensitivity, specificity, positive predictive value (PPV), and negative predictive value (NPV).

## Results

### Cohort Characteristics

Of 2529 patients, 125 patients had recorded FC values at least 60 days after but within one year of surgery; 37 (30%) were categorized as either low-risk or high-risk and received biologic prophylaxis and had postoperative colonoscopy (Table 1). A total of 15 (41%) had initial FC values < 50 ug/g, and 22 (59%) had values ≥ 50 ug/g. Median age at the time of surgery was 36 (IQR 25–48). Anti-TNF therapy was the most common biological prophylaxis. Median time to first FC was 217 days (IQR 131–288), similar between FC < 50 ug/g and FC ≥ 50 ug/g (193 days vs. 217 days, *P* = .72). Median time to first colonoscopy was 234 days (189–369). 21 (57%) patients had 1 + subsequent colonoscopy, 6 (40%) in FC < 50 ug/g versus 15 (68%) in FC ≥ 50 ug/g, *P* = .11. Median number of subsequent colonoscopies, 0 (IQR 0–1) versus 1 (IQR 0–2), *P* = .18, as well as time to subsequent colonoscopy, 375 versus 373 days, *P* = .7, was similar in both cohorts. There is no significant association between initial FC value and risk group. Available follow-up time was longer in those with initial FC ≥ 50 ug/g compared to < 50 ug/g (1055 vs. 599 days, *P* = .01).

**Table 1.** Demographics and comparison of FC groups

	Total *n* = 37		
Median surgery age	36 (25–48)		
Low-risk (*n*, %)	20 (54)		
On biologic	3 (15)		
High-risk on biologic (*n*, %)	17 (46)		
Anti-TNF	11 (65)		
Ustekinumab	4 (23)		
Vedolizumab	2 (12)		
Median time to first FC, days	217 (131–288)		
Median time to first colonoscopy, days	234 (189–369)		
At least 1 subsequent colonoscopy (*n*, %)	21 (57)		

	FC < 50 ug/g (*n* = 15)	FC ≥ 50 ug/g (*n* = 22)	*P*-value
Low-risk	7 (47)	13 (59)	.51
High-risk received prophylaxis	8 (53)	9 (41)	
Median time to first FC, days	193 (109–287)	217 (135–298)	.72
Median time to first colonoscopy, days	237 (202–377)	230 (185–354)	.85
Median # subsequent colonoscopies	0 (0–1)	1 (0–2)	.11
Median time to subsequent colonoscopy, days	375 (193–591)	373 (245–453)	.7
Median time to endoscopic recurrence, days	-	145 (56–217)	n/a
Ever endoscopic recurrence (*n*)	0	9	.006
No endoscopic recurrence (*n*)	17	16	
Rutgeerts within 1 year of FC			n/a
i0	6 (60)	4 (25)	
i1	3 (30)	4 (25)	
i2a	1 (10)	1 (6.3)	
i2b	-	5 (31)	
i3	-	1 (6.3)	
i4	-	1 (6.3)	
Median time to surgical recurrence, days	-	1416 (839–1677)	n/a
Ever surgical recurrence (*n*)	0	3	.26
No surgical recurrence (*n*)	17	22	
Median follow-up time, days	599 (410–744)	1055 (577–2017)	.01

Categorical variables are given as numbers (percentage). Nonparametric continuous variables are given as median (IQR). Fisher exact tests were utilized to calculate *P*-values for categorical variables and Student’s *T*-test was utilized for continuous variables. Anti-TNF, antitumor necrosis factor; FC, fecal calprotectin.

### Recurrence

Compared to patients with FC ≥ 50 ug/g, FC < 50 ug/g had significantly less endoscopic recurrence over the available follow-up period (0% vs. 36%, *P* = .006; Table 1). Median time from initial FC to first endoscopic recurrence in the ≥50 ug/g cohort was 145 days. Rutgeerts scores recorded within one year of the initial FC are shown in Table 1. There was no significant difference in rates of surgical recurrence (0% vs. 12%, *P* = .26). Median time from initial FC to surgical recurrence in the ≥50 ug/g cohort was 1416 days.

### Test Characteristics

There were 39 matched pairs of FC and colonoscopy. At an FC cutoff of 50 ug/g, calculated sensitivity was 90% and NPV was 93%, whereas specificity and PPV were 48% and 38%, respectively. Of 3 patients considered low-risk for recurrence at baseline and on postoperative biologic prophylaxis, all had initial FC < 150 ug/g and no endoscopic recurrence in the follow-up period.

### Serial FC

Seventeen patients had at least 2 recorded FC values within 36 months of the initial measurement. Supplementary Figure S1 shows the trend of FC in patients who were initially in endoscopic remission and remained in remission. Interval FC corresponds to the nearest interval colonoscopy. Those who were initially in remission and remained so generally had decreasing FC values. The number of patients in initial remission who then experienced recurrence, those in initial recurrence who then experienced remission, and those in initial recurrence who remained in recurrence did not have sufficient N.

## Discussion

The results from this real-world, multicenter, retrospective analysis support the use of an FC cutoff of 50 ug/g in a low-risk, postoperative CD population as proposed by AGA guidelines. With high sensitivity and NPV, this aligns with the false negative rate of 1.4% reported within the guidelines and underscores its utility in the avoidance of unnecessary endoscopy. Lower specificity and PPV are unsurprising given the low pretest probability of recurrence in this cohort. Those with a FC < 50 ug/g had lower rates of overall endoscopic recurrence over the follow-up period as well as no surgical recurrence, which may suggest a longer predictive value, although this should be interpreted with caution given our small sample size and various lengths of follow-up. All 3 patients considered low-risk at baseline and on biologic prophylaxis had initial FC < 150 ug/g without subsequent endoscopic recurrence, though sample size again limits interpretation of this cutoff.

The trend of FC may be important, as those who had decreasing values maintained endoscopic remission, though sample size limits our ability to ascertain meaningful long-term outcomes from these results. Understanding the trend in those who are initially in remission and subsequently recur, as well as those who initially have active inflammation that subsequently remits, is of additional interest for the management of these patients and should be investigated with a larger cohort.

There are several limitations to this study. The most significant limitation as previously mentioned is sample size, and while results may support the real-world application of proposed guidelines, they may not be sufficient to draw conclusions regarding FC and post-surgical outcomes. This was a retrospective analysis and subject to inherent biases. Though the initial cohort included a large representative population, few utilized FCs in the postoperative period. This may in part be due to including surgical dates spanning between 2009 and 2020 and heterogeneity in postoperative management practices over time. Patients without colonoscopies within the designated time interval were excluded, which may introduce bias. Supplementary Table S1 with descriptive information on these patients is included. The number of postoperative colonoscopies was similar, but follow-up periods significantly differed between groups. Because modified Rutgeerts scores could be retrospectively assigned, this may introduce bias as clinicians were not blinded to the clinical status of the patient. However, this was required in < 5% of cases of the original cohort. Our results need to be validated in larger, prospective populations and over longer periods of time to ensure optimal patient selection for these management approaches.

## Supplementary Material

otae016_suppl_Supplementary_Material

## Data Availability

Data is available in supplementary material.

## References

[1] Mosli MH , Zou G, Garg SK, et al. C-reactive protein, fecal calprotectin, and stool lactoferrin for detection of endoscopic activity in symptomatic inflammatory bowel disease patients: a systematic review and meta-analysis. Off J Am Coll Gastroenterol. 2015;110(6):802-819. 10.1038/ajg.2015.120 25964225

[2] Plevris N , Lees CW. Disease monitoring in inflammatory bowel disease: evolving principles and possibilities. Gastroenterology. 2022;162(5):1456-1475.e1. 35101422 10.1053/j.gastro.2022.01.024

[3] Patwala K , De Cruz P. Postoperative Crohn’s disease. Biomarkers in Inflammatory Bowel Diseases. Springer International Publishing; 2019:89-97.

[4] Tham YS , Yung DE, Fay S, et al. Fecal calprotectin for detection of postoperative endoscopic recurrence in Crohn’s disease: systematic review and meta-analysis. Therap Adv Gastroenterol. 2018;11:1756284818785571. doi: 10.1177/1756284818785571 PMC6048608 30034529

[5] Ananthakrishnan AN , Adler J, Chachu KA, et al.; AGA Clinical Guidelines Committee. Electronic address: clinicalpractice@gastro.org. AGA clinical practice guideline on the role of biomarkers for the management of Crohn’s disease. Gastroenterology. 2023;165(6):1367-1399. 37981354 10.1053/j.gastro.2023.09.029

[6] Bachour SP , Shah RS, Lyu R, et al. Mild neoterminal ileal post‐operative recurrence of Crohn’s disease conveys higher risk for severe endoscopic disease progression than isolated anastomotic lesions. Aliment Pharmacol Ther. 2022;55(9):1139-1150. 35285534 10.1111/apt.16804 PMC9677520

