# Supplementary material for: American Gastroenterological Association-Proposed Fecal Calprotectin Cutoff of 50 ug/g is Associated With Endoscopic Recurrence in a Real-World Cohort of Patients With Crohn’s Disease Post-ileocolic Resection
Source: Crohns Colitis 360. 2024 Mar 9;6(1):otae016. doi: 10.1093/crocol/otae016 (PMC10960600; doi:10.1093/crocol/otae016)
Supplement: otae016_suppl_Supplementary_Material [file otae016_suppl_supplementary_material.docx]

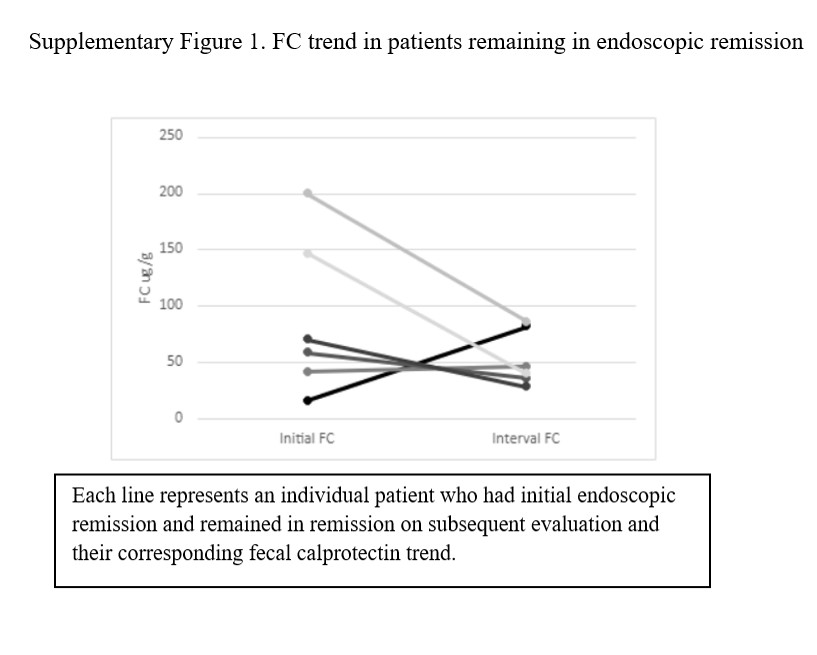


Supplementary Table 1. Patients excluded due to no post-operative colonoscopy

|  | Total n = 17 | | |
| --- | --- | --- | --- |
| Median surgery age (IQR) | 41 (37 – 57) | | |
| Low-risk (n, %)  On biologic  High-risk on biologic (n, %)  Anti-TNF  Ustekinumab  Vedolizumab | 13 (76)  0  4 (24)  2 (50)  2 (50)  0 | | |
| Median time to first FC, days (IQR)  Median first FC (IQR) | 148 (111 –229)  76 (27 – 114) | | |
|  | FC <50ug/g (n = 5) | FC ≥ 50ug/g (n=12) | P-value |
| Low-risk  High-risk received prophylaxis | 3 (60)  2 (40) | 10 (83)  2 (17) | 0.53 |
| Median time to first FC, days | 148 (131 – 189) | 149 (95 – 229) | 0.3 |
| Ever surgical recurrence | - | - | n/a |
| Categorical variables are given as numbers (percentage). Nonparametric continuous variables are given as median (IQR). Fisher exact tests were utilized to calculate p-values for categorical variables and Student’s T-test was utilized for continuous variables. Anti-TNF, anti-tumor necrosis factor; FC, fecal calprotectin. | | | |
